# Comparison of mannitol and methacholine to predict exercise-induced bronchoconstriction and a clinical diagnosis of asthma

**DOI:** 10.1186/1465-9921-10-4

**Published:** 2009-01-23

**Authors:** Sandra D Anderson, Brett Charlton, John M Weiler, Sara Nichols, Sheldon L Spector, David S Pearlman, A305 Study Group

**Affiliations:** 1 Department of Respiratory and Sleep Medicine, Royal Prince Alfred Hospital, Missenden Road, Camperdown, NSW 2050, Australia; 2 Pharmaxis Ltd, 2/10 Rodborough Rd, Frenchs Forest, NSW 2086, Australia; 3 CompleWare Corporation, PO Box 3090, Iowa City, IA 52244-3090 and University of Iowa, Iowa City, IA 52242, USA; 4 CompleWare Corporation, PO Box 3090, Iowa City, IA 52244-3090, USA; 5 California Allergy and Asthma Medical Group, 11645 Wilshire Blvd., Ste. 1155, Los Angeles, CA 90025, USA; 6 Colorado Allergy & Asthma Centers, PC, 125 Rampart Way, Suite 150, Denver, CO 80230-6405, USA

## Abstract

**Background:**

Asthma can be difficult to diagnose, but bronchial provocation with methacholine, exercise or mannitol is helpful when used to identify bronchial hyperresponsiveness (BHR), a key feature of the disease. The utility of these tests in subjects with signs and symptoms of asthma but without a clear diagnosis has not been investigated. We investigated the sensitivity and specificity of mannitol to identify exercise-induced bronchoconstriction (EIB) as a manifestation of BHR; compared this with methacholine; and compared the sensitivity and specificity of mannitol and methacholine for a clinician diagnosis of asthma.

**Methods:**

509 people (6–50 yr) were enrolled, 78% were atopic, median FEV_1 _92.5% predicted, and a low NAEPPII asthma score of 1.2. Subjects with symptoms of seasonal allergy were excluded. BHR to exercise was defined as a ≥ 10% fall in FEV_1 _on at least one of two tests, to methacholine a PC_20 _≤ 16 mg/ml and to mannitol a 15% fall in FEV_1 _at ≤ 635 mg or a 10% fall between doses. The clinician diagnosis of asthma was made on examination, history, skin tests, questionnaire and response to exercise but they were blind to the mannitol and methacholine results.

**Results:**

Mannitol and methacholine were therapeutically equivalent to identify EIB, a clinician diagnosis of asthma, and prevalence of BHR. The sensitivity/specificity of mannitol to identify EIB was 59%/65% and for methacholine it was 56%/69%. The BHR was mild. Mean EIB % fall in FEV_1 _in subjects positive to exercise was 19%, (SD 9.2), mannitol PD_15 _158 (CI:129,193) mg, and methacholine PC_20 _2.1(CI:1.7, 2.6)mg/ml. The prevalence of BHR was the same: for exercise (43.5%), mannitol (44.8%), and methacholine (41.6%) with a test agreement between 62 & 69%. The sensitivity and specificity for a clinician diagnosis of asthma was 56%/73% for mannitol and 51%/75% for methacholine. The sensitivity increased to 73% and 72% for mannitol and methacholine when two exercise tests were positive.

**Conclusion:**

In this group with normal FEV_1_, mild symptoms, and mild BHR, the sensitivity and specificity for both mannitol and methacholine to identify EIB and a clinician diagnosis of asthma were equivalent, but lower than previously documented in well-defined populations.

**Trial registration:**

This was a multi-center trial comprising 25 sites across the United States of America. (NCT0025229).

## Background

Asthma can be difficult to diagnose and no single symptom or test should be used in isolation to make the diagnosis. A correct diagnosis is important in order for patients to receive appropriate therapy [1]. Because bronchial hyperresponsiveness (BHR) is a hallmark of asthma, bronchial provocation challenge tests (BPTs) with a variety of agents have been used to assist in its diagnosis [2-4].

Methacholine is a commonly used agent, delivered as a wet aerosol. Methacholine acts directly on acetylcholine receptors on smooth muscle causing contraction and airway narrowing. Methacholine has been reported to have a high sensitivity to identify BHR and a negative test is often used to exclude asthma [5,6].

Provocation tests that use indirect stimuli (e.g. exercise and mannitol) have a high specificity for asthma [7] causing smooth muscle contraction by release of endogenous mediators including prostaglandins, leukotrienes, and histamine [8,9]. Evaporative water loss occurs in conditioning the inspired air and causes exercise induced bronchoconstriction (EIB) by inflammatory mediators of mast cell origin [10,11]. Exercise is generally recognised as having a low sensitivity to identify BHR; EIB is consistent with a diagnosis of asthma [12] and responds to chronic treatment with inhaled corticosteroids (ICS) and other drugs used in the treatment of asthma [13-15].

A dry powder of mannitol has been developed as an indirect BPT [16] and is available as a standardized test kit. The test kit contains pre-filled mannitol capsules in escalating doses and a hand-held dry powder inhaler device. Safety and efficacy of mannitol as a BPT were established in a large Phase III clinical trial in patients with asthma and in healthy subjects [7].

The usefulness of mannitol as a BPT in subjects with signs and symptoms of asthma but no clear diagnosis has not been investigated previously. The aim was to study a large population of subjects to compare the sensitivity and specificity of mannitol with methacholine to detect EIB as a manifestation of BHR and to identify a clinician diagnosis of asthma.

### Subjects: inclusion criteria

Subjects aged 6–50 years (BMI < 35) with signs and symptoms suggestive of asthma according to the National Institute of Health (NIH) Questionnaire [17] but without a firm diagnosis of asthma or an exclusion of the diagnosis of asthma (e.g. had an equivocal diagnosis of asthma or had been referred for further investigation of asthma-type symptoms) were included. Subjects had at least Step 1 symptoms according to the NAEPPII asthma severity grading (symptoms ≤ 2 times per week; asymptomatic between exacerbations; exacerbations of only a few hours to a few days; and night time symptoms of ≤ 2 times per month). They were required to have an FEV_1 _≥ 70% of the predicted value at the Screening Visit [18,19].

Subjects were excluded from participating in this study if they: had any known other pulmonary disease; had smoked more than 1 cigarette per week within the past year or had a ≥ 10 pack year smoking history; had a respiratory tract infection within the previous 4 weeks; had been skin test positive to aeroallergens that were present in the environment during the time of enrolment and reported worsening of symptoms when exposed to these aeroallergens during the study; had been diagnosed at Screening Visit as definitively having asthma (95 to 100% likelihood) or not having asthma (0 to < 5% likelihood); had clinically significantly abnormal chest x-ray or ECG; or had failed to observe washout of medications that would interfere with BPT (including, but not limited to, no use of corticosteroids within 4 weeks of the Screening Visit).

## Methods

The protocol was approved by a central institutional review board. Each subject or parent gave informed consent in writing. The study consisted of 5 visits to the clinic. On the Screening Visit the following were assessed: eligibility; demographic data; medical history; medications; spirometry with reversibility (following 360 mcg of albuterol); allergy skin test reactivity to common allergens (positive test taken as 3 mm wheal). The NIH NAEPPII questionnaire was answered and a score was assigned [20]. Vital signs including blood pressure, heart rate, and respiratory rate were measured in the sitting position and an ECG performed. Based on this information, a respiratory physician assigned one of 6 diagnoses at this visit on the basis of the likelihood of asthma as follows: asthma is extremely likely or definite (95%–100% likelihood); asthma is very likely (72.5 to < 95%); asthma is probable (50 to < 72.5%); asthma is possible (27.5 to < 50%); asthma is unlikely but cannot be excluded (5 to 27.5%); and asthma is very unlikely (0-<5%). Those with 5 to 95% likelihood were included in the study.

Visit 2 occurred 1–4 days after Visit 1 and within 2 hrs of the time of Screening. Adverse events, medications, and withholding times were reviewed (Table 1), and spirometry measured to confirm values on the screening day. This was followed by a brief physical examination. Exercise was performed with vital signs being measured before and after exercise. At Visit 3, the procedures were the same as Visit 2 and occurred within 1–4 days. At Visit 4, adverse events and medications were reviewed, withholding times were checked, and spirometry was performed to confirm FEV_1 _was within 15% of Visit 1. The challenge agent was either mannitol or methacholine, and the choice was randomly determined. The time of the test was documented for each challenge. Vital signs were measured in the sitting position before and after the challenge test. Cough and pulse oximetry were recorded during mannitol challenges. Full spirometry was measured before and at 15 minutes after completion of the mannitol challenge with FEV_1 _only being performed after each dose. ECG was performed before and after mannitol challenge. Visit 5 was a repeat of the procedures of Visit 4 with the reciprocal challenge being administered (either mannitol or methacholine). A respiratory physician then assigned one of the diagnoses of likelihood of asthma evaluated at the Screening Visit, above. The NAEPII asthma severity grading score was also re-evaluated at Visit 5 but not necessarily by the same physician.

**Table 1:** Medications and other factors that may decrease bronchial hyperresponsiveness and their required withholding periods

	**FACTOR**	**Withholding Period**
**Inhaled agents**	Short acting bronchodilators (isoproterenol, isoetharine, metaproterenol, albuterol, levalbuterol, terbutaline) (e.g. Proventil^® ^or Ventolin^®^)	8 hr
	
	Inhaled anticholinergics or combination products (e.g. Atrovent^® ^or Combivent^®^)	1 week
	
	Medium acting bronchodilators (ipratropium)	1 week
	
	Long acting inhaled bronchodilators (salmeterol, formoterol) (e.g. Serevent^® ^or Foradil^®^)	2 weeks
	
	Inhaled corticosteroid/long acting inhaled bronchodilator combination (e.g. Advair^®^)	4 weeks

**Oral bronchodilators**	Theophylline	24 hr
	
	Intermediate theophylline	48 hr
	
	Long acting theophylline	48 hr
	
	Standard β-agonist tablets	24 hr
	
	Long acting β-agonist tablets	48 hr

**Corticosteroids**	There is no washout for topical steroids applied to skin unless they are high potency steroids	4 weeks

**Other medications**	Hydroxyzine, cetirizine (and other antihistamines)	72 hr
	
	Tiotropium bromide	72 hr
	
	Nasals steroids	1 week
	
	β-blockers	1 week
	
	Cromolyn sodium	2 weeks
	
	Nedocromil	2 weeks
	
	Leukotriene modifiers	6 weeks

**Foods**	Coffee, tea, cola drinks, chocolate (caffeinated foods)	12 hr

**Strenuous exercise or exposure to cold air to a level that would be expected to interfere with challenges**		12 hr

**Tobacco**		6 hr

### Bronchial provocation tests

#### Exercise challenge

Exercise was performed by running on a motorised treadmill whilst breathing medical grade dry air (20–25°C) from a Douglas Bag [14]. Briefly exercise was ramped up over two minutes to 80–90% predicted heart rate (220-age) and then sustained for 6 minutes. The highest FEV_1 _was measured before and at 5, 10, 15 and 30 minutes after exercise. The % fall in FEV_1 _was calculated by subtracting the lowest value recorded after exercise (best of two acceptable attempts at each time point) from the value measured immediately before exercise and expressing it as a percentage of the pre exercise value. A subject was deemed positive if there was a fall of ≥ 10% in FEV_1 _at one time point [2,3] on at least one of the two exercise challenges.

#### Mannitol challenge

The mannitol test was carried out as per the standard laboratory protocol for this challenge test using the commercially available mannitol test kit (known as Aridol™ or Osmohale™ Pharmaxis Ltd, AUS) [7]. The FEV_1 _was measured 60 seconds after each mannitol dose (0, 5, 10, 20, 40, 80, 160, 160, 160 mg). The subjects were asked to exhale completely before taking a controlled deep inspiration from the device and to hold their breath for 5 seconds then exhale through their mouth before removal of the nose clip. Sixty seconds after inhalation of the 0 mg capsule the FEV_1 _was measured in duplicate. The highest of these values was taken as the baseline FEV_1 _and was used to calculate the target FEV_1 _value that indicated a 15% fall in response to the mannitol challenge. The test result expressed is a PD_15_.

The procedure outlined above for the 0 mg capsule was repeated for each dose step until a 15% fall in FEV_1 _was achieved (or a 10% fall between consecutive doses) or the cumulative dose of 635 mg had been administered.

#### Methacholine

Methacholine (Provocholine™, Methapharm CA) was delivered from a nebulizer (DeVilbiss model 646) by the dosimeter method [2]. The concentrations were: 0.0312, 0.0625, 0.125, 0.25, 0.5, 1, 2, 4, 8, 16 mg/mL. Each concentration required five inhalations from functional residual capacity to total lung capacity. Spirometry was performed within 3 minutes. The response to methacholine was expressed as the concentration required to provoke a 20% fall in FEV_1 _from the pre-challenge value (PC_20_).

#### Bronchodilator

On Visit 1 a dose of 360–400 mcg of albuterol (salbutamol) was administered and the FEV_1 _measured between 15 and 30 minutes. A positive test was defined as a 12% increase in FEV_1 _above the baseline value.

#### Statistical testing

Results were expressed using univariate statistics including means, standard deviations, ranges, and medians. Mannitol and methacholine responses were log transformed for calculation of the geometric means. The analysis plan specified a test of non-inferiority to be achieved if the lower 95% credible limit for the adverse-event-free-ratio (1-AER_mannitol_)/(1-AER_methacholine_) exceeded 0.80.

#### Analysis

The sensitivity and specificity of a mannitol positive test (defined as ≥ 15% fall in FEV_1 _≤ 635 mg or a ≥ 10% fall in FEV_1 _between consecutive doses) and a methacholine positive test (defined as PC_20_FEV_1 _≤ 16 mg/ml) with respect to EIB (defined as ≥ 10%, 15% or 20% fall in FEV_1 _after a standardized treadmill run) and with respect to a clinician's diagnosis of asthma at Visit 5 were calculated. Additional analyses were performed excluding those with a mannitol test taking longer than 35 minutes because the osmotic gradient will not progressively increase if the time between doses is prolonged.

#### Safety data

Vital signs (including blood pressure, heart rate, and respiratory rate) using the intent-to-treat population (ITTP, n = 391) and their changes during challenge tests are described. The adverse events were tabulated following each challenge test. The per-protocol population (PPP, n = 375) included all subjects with no major protocol violations who completed all of the required challenge tests, including methacholine and mannitol challenges.

In the protocol cough was identified as an adverse event if it prevented the challenge from being completed in which case it was documented as severe at the time of testing.

#### Blinding

A respiratory physician was to make the Clinician diagnosis at the final visit (Visit 5) with access to the data on the exercise challenges, history, examination, skin tests, and FEV_1 _reversibility but not the mannitol and methacholine challenge test result. Site staff members performing mannitol and methacholine challenges were not provided with other diagnostic information about the subject to assure that there was no bias in the performance of these tests. Mannitol and methacholine challenges were given in a restricted randomization scheme that produced equal numbers of each order.

To accomplish proper blinding, the mannitol and methacholine challenge team did not have access to the electronic case report form (eCRF) or to other physiological data. Similarly, other site personnel did not have access to the mannitol and methacholine challenge data. Mannitol and methacholine challenge data were able to be reviewed by the Data Manager at CompleWare Inc but they were only provided to the Sponsor (Pharmaxis Ltd) at the end of the study.

## Results

**Figure 1:**
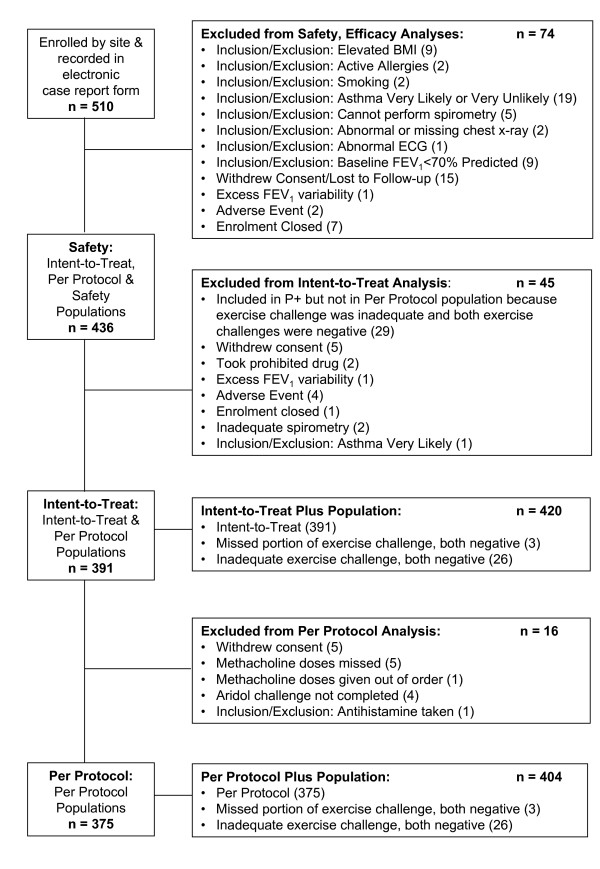
**Subject disposition**.

The disposition of the subjects is given in Figure 1. There were 16 people in the ITTP who were not included in the PPP. The data are given for the PPP unless otherwise stated because it represents the results for all the subjects who performed all the tests. The age distribution for 375 subjects in the PPP are presented in Table 2. The characteristics of the subjects are summarised in Table 3. There were 96 children and 279 adults (> 18 years); 51.5% female; 76.3% Caucasian, 8.3% Hispanic and 8.5% Black; with near-normal baseline spirometry; with low NAEPPII asthma score of 1.2 (SD 0.5); 78% atopic; and 7.5% responding positively to a bronchodilator. Of the 375, 145 were considered by the respiratory physician to have a 50% or more likelihood of having asthma at Visit 1.

**Table 2:** Demographics: Age distribution in the intent-to-treat, in the excluded and safety, and in the per-protocol populations.

	**Intent-to-Treat**		**Excluded and Safety**		**Per-Protocol**	
**Age**	**Number**	**Percent**	**Number**	**Percent**	**Number**	**Percent**

**Total**	**391**		**509**		**375**	

6–7	6	1.5%	9	1.8%	6	1.6%

8–9	7	1.8%	11	2.2%	7	1.9%

10–11	20	5.1%	25	4.9%	19	5.1%

12–15	38	9.7%	48	9.4%	36	9.6%

16–18	44	11.3%	57	11.2%	43	11.5%

19–24	113	28.9%	135	26.5%	108	28.8%

25–30	69	17.6%	84	16.5%	68	18.1%

31–35	28	7.2%	44	8.6%	25	6.7%

36–40	31	7.9%	44	8.6%	29	7.7%

41–45	19	4.9%	25	4.9%	19	5.1%

46–50	16	4.1%	27	5.3%	15	4.0%

**Table 3:** Anthropometric data, forced expiratory volume in one second, and smoking history in the per protocol population.

	**Age (yr)**	**BMI**	**FEV_1 _(L)**	**% Pred FEV_1_**	**Post BD FEV_1 _(L)**	**Pack Yrs**	**Ht (cm)**	**Wt (kg)**
**Mean**	24.3	24.4	3.32	93.6%	3.48	2.9	167.4	69.2

**SD**	10.2	4.5	0.82	10.0	0.87	2.8	13.1	18.4

**Range**	6–50	13.4–34.9	1.15–5.62	63.7–140.1	1.29–5.92	0–9	118–204.5	20–135.2

**Median**	22	24.1	3.24	93.3	3.32	2.5	167.6	69

There were 163 (43.5%) subjects who had a positive response to exercise (Exc+) with a ≥ 10% fall in FEV_1 _on at least one test; 119 recording this on the first exercise test with 66 of these 119 recording a ≥ 15% fall in FEV_1_. There were 168 (44.8%) subjects with a positive test to mannitol (Mann+). Of these, 109 achieved a 15% fall in 635 mg and the remaining achieved a positive test by a 10% fall in FEV_1 _between consecutive doses. Seventy three percent achieved this response within the first 6 doses of mannitol (≤ 315 mg). There were 156 (41.6%) with a positive test to methacholine (Mech+) with a PC_20 _≤ 16 mg/ml, and 26% achieved this response within the first 6 concentrations (≤ 1 mg/ml) being delivered. The percentile values and median data for the positive responses are given in Table 4.

**Table 4:** Percentile and geometric mean values for bronchial provocation tests as well as maximum % fall for subjects with positive exercise challenges.

	**Percentiles**			
		
	**25th**	**50th**	**75th**	**Geomean (95% CI)**
**Mann^+ ^PD_15 _(mg)**	72	234	374	158 (129,193)

**Mech^+ ^PC_20 _(mg/ml)**	0.84	2.98	6.53	2.12 (1.7,2.64)

**Exc^+ ^% Fall**	23.6%	15.5%	12.4%	19.1% (9.25)*

*Mean (SD)

**Figure 2:**
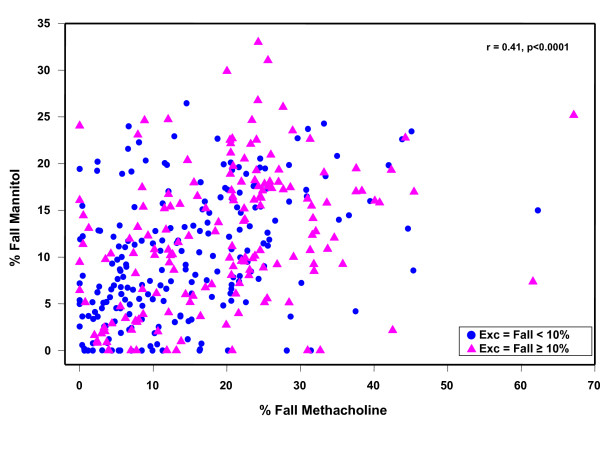
**The maximum percentage fall in FEV_1 _for mannitol and methacholine in subjects in the per-protocol population**.

Sensitivity and specificity of mannitol and methacholine to identify a subject with different levels of severity of EIB is given in Table 5. There was no significant difference between mannitol and methacholine to identify EIB; however, these agents did not necessarily identify the same people and the agreement between the mannitol and methacholine test results was 69%. Agreement for mannitol and exercise was 62% and for methacholine and exercise was 63%. The relationship between the reactivity to mannitol expressed as log RDR and the reactivity to exercise expressed as the maximum % fall in FEV_1 _was significant but not strong (r = 0.256, p < 0.001, n = 312). Maximum % fall in FEV_1 _to mannitol in relation to methacholine (r = 0.41 p < 0.0001) in those positive and negative to exercise is illustrated in Figure 2. Forty-six (29%) people had a fall ≥ 30% in FEV_1 _after methacholine, 2 (1.2%) after mannitol and 25 (15.3%) after exercise. The mean maximum % fall (SD) in FEV_1 _after those with a positive methacholine challenge was 27.7% ± (8.2) and after a positive mannitol challenge was 16.1% ± (5.6). The fall after mannitol was 19.2% ± (4) excluding those who were positive due to a 10% fall between doses, and this was the same as the fall after exercise19.1% (9.4). Sensitivity and specificity of mannitol and methacholine to identify Exc+ 10% in relation to symptoms score is summarised in Table 6.

**Table 5:** Sensitivity and specificity of challenge at different cut points for a positive test.

**Exercise Positive Cut-Points – % fall from baseline**		**10%**	**15%**	**20%**
Mannitol	Sensitivity	58.6	69.4	78.6
n = 372	Specificity	65.2	62.0	60.8

Excluding those with challenge > 35 min	Sensitivity	64.1	75.3	82.7
n = 319	Specificity	59.9	57.0	55.4

Methacholine 16 mg/ml	Sensitivity	55.2	67.4	80.3
n = 375	Specificity	68.9	66.1	65.2

**Table 6:** Sensitivity and specificity to identify exercise-induced bronchoconstriction (EIB) in relation to the asthma severity score.

		**NAEPPII**							
		
		**Visit 1**				**Visit 5**			
Asthma Severity Score		0	1	2	3	0	1	2	3

	N mann =	0	308	47	17	13	294	48	15
	N mech =	0	310	48	17	14	296	48	15

Mannitol	Sensitivity		56.6	75.0	60.0	50.0	55.1	78.3	66.7
635 mg	Specificity		69.2	48.4	42.9	72.7	67.7	56.0	16.7

Methacholine	Sensitivity		54.7	62.5	50.0	0.0	56.3	56.5	44.4
16 mg/ml	Specificity		72.8	46.8	71.4	66.7	70.8	56.0	66.7

**Figure 3:**
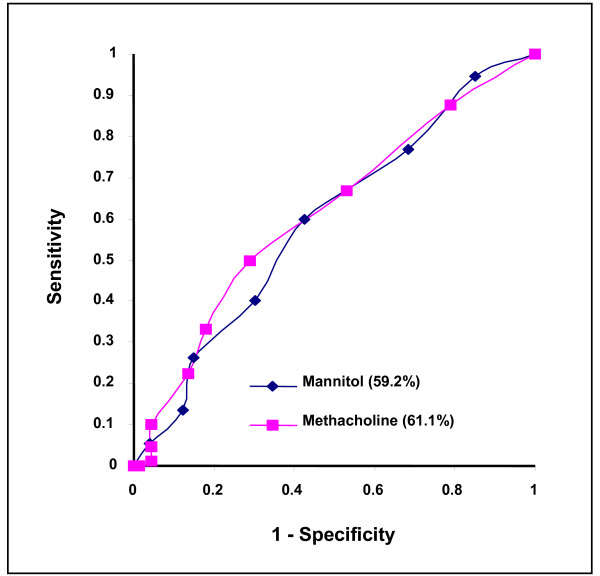
**The receiver operating curve for mannitol and methacholine to identify exercise-induced bronchoconstriction defined as ≥ 10% fall in FEV_1 _from baseline at one time point after exercise on at least one of two exercise tests**.

Mannitol had a sensitivity of 67% to identify a Mech+ PC_20 _of ≤ 16, 68% for ≤ 12, and 68% for ≤ 8 mg/ml and a sensitivity of 77% to identify ≤ 4 mg/ml and 83% to identify ≤ 2 mg/ml. Methacholine had a sensitivity of 62% for identifying a Mann+ response. An Exc+10% on the first test had a sensitivity of 62% to identify Exc+10% on the second test. The receiver operator curves for mannitol and methacholine in relation to identifying EIB is illustrated in Figure 3 and are almost identical for mannitol and methacholine. The negative and positive predictive values for mannitol and methacholine were close generally differing by 0.01 or less. Similarly the negative (0.64 and 0.65) and positive likelihood ratios (1.71 and 1.79) were similar for mannitol and methacholine respectively.

There were 28 (7.5%) subjects in the per protocol population who reversed at least 12% after a beta_2 _agonist. This small group showed a sensitivity of mannitol and methacholine to identify EIB of 68.4% and 73.7% and a specificity of 44.4% and 55.5% respectively.

Sensitivity and specificity for mannitol and methacholine to identify a 10% fall after exercise in children and a clinical diagnosis at Visit 5 is given in Table 7. Of the 375 PPP, there were 240 (64%) identified as having a clinical diagnosis of asthma at Visit 5 (ClinDx5+) and 48% of these had a likelihood of asthma (see definitions above) greater than 50% assigned at Visit 1. Fifteen percent of the subjects who received a clinical diagnosis of asthma were negative to all three challenges. Of the 135 who did not receive a clinical diagnosis of asthma at Visit 5 (ClinDx5-), 78% had a likelihood of asthma of less than 50% assigned at Visit 1. Sensitivity and specificity of mannitol to predict ClinDx5+ was 55% and 73%. The sensitivity for mannitol rose to 73% when ClinDx5+ was associated with two Exc+ 10% tests. The comparable sensitivity and specificity for methacholine (PC_20 _≤ 16 mg/ml) was 51% and 75% and sensitivity rose to 72% when ClinDx5+ was associated with two Exc+10% tests.

**Table 7:** Children < 18 yrs (n = 115). Per protocol population

**"Gold Standard"**		**10% Ex^+^**	**ClinDx5^+^**
Mannitol	Sensitivity	60.1	63.2
	Specificity	58.5	81.4

Methacholine 16 mg/ml	Sensitivity	70.0	66.2
	Specificity	54.5	62.9

The positive and negative predictive values for mannitol for a ClinDx5+ were 79% and 48% and for methacholine they were 78% and 46%. There were 106 subjects negative to mannitol, 118 negative to methacholine and 92 negative to exercise who were given a clinician diagnosis of asthma at Visit 5. When the subjects who took longer than 35 min for a mannitol challenge were excluded, the sensitivity of mannitol to identify a 95% risk of having asthma as judged by the clinician was 76%. For methacholine this equivalent value was 67%.

The time to perform a positive mannitol test was significantly shorter than a positive methacholine test by approximately 25 min (19.9 min [SE 0.9] vs 44.5 min [SE 1.4]). In those Mech+ only it was 48.3 min (SE 1.7) and for those Mann+ only it was 19.2 min (SE 1.3).

Time for recovery of FEV_1 _to 95% of baseline was similar for all tests. Mannitol was 21.6 min (SD 9.0) vs. methacholine 21.06 min (SD 6.96); the maximum time for recovery on the two exercise tests was 22.9 min (SD 13.7). Median time for recovery after mannitol and methacholine was 19 min (interquartile range 17–24).

Sensitivity and specificity for methacholine to identify EIB were considerably less consistent across the centres than for mannitol. The between centre standard deviation for the log odds ratio for methacholine and mannitol was respectively 1.26 vs. 0.32 for sensitivity (p < 0.02) and 0.68 vs 0.47 (p = NS) for specificity. Thus there was significantly less variation in sensitivity of mannitol to identify EIB between centres compared with methacholine.

The skin test results for the wide variety of allergies are summarised in Table 8 for the per protocol population. The percentage of subjects positive to one or more skin tests for those Exc+ was 42.6%, for Mann+ it was 47.1% and for Mech+ it was 41.8%. It was higher in those achieving a ClinDx5+ 63.0%.

**Table 8:** The frequency of positive skin tests (> 3 mm wheal) to a variety of allergens in the per protocol population (n = 375).

	**Cat**	**Cockroach**	**Dog**	**Grass**	**House Dust Mites**	**Mold**	*Cedar	**Rag weed**	**Other Weeds**	**Trees**
**Positive**	153	48	75	180	175	99	56	106	126	146
**Tests done**	363	299	363	336	347	363	145	298	323	328
**% Positive**	42.1	16.1	20.7	53.6	50.4	27.3	38.6	35.6	39.0	44.5

*Mountain Cedar

During mannitol challenges, frequency of cough was monitored in 419 subjects. Of these 391 had cough (93%) with 204 having occasional cough (49%) that did not interfere with the challenge, 178 frequent cough (42%) that delayed the administration of the next dose and 9 severe cough (2.1%) that caused the challenge to be stopped and an adverse event to be recorded.

Vital signs changed as expected, with increased in systolic and diastolic blood pressure, heart rate, and respiratory rate associated with both exercise challenges. Trivial increases in blood pressure were seen associated with mannitol and methacholine challenges. Oxygen saturation did not change appreciably with mannitol challenge and only 3 subjects had > 3% reduction. Heart rate increased slightly after mannitol challenge and decreased slightly after methacholine challenge. Respiratory rate was also largely unchanged with both mannitol and methacholine challenge.

### Adverse events

All adverse events which commenced after the challenges were reported by MedDRA System Organ Class and Preferred Term and tabulated (Table 9) There were more adverse events on mannitol compared with methacholine 130 vs 89. The distribution across range of severity of events was the same. There were 62 moderate adverse events on mannitol and 35 on methacholine, with 9 severe ones on mannitol and 3 on methacholine. There were no serious adverse events for any of the challenge tests. Mannitol was non-inferior to methacholine in the sense that the proportion of subjects who did not experience adverse events in the mannitol challenge was at least 80% of the proportion who did not experience adverse events in the methacholine challenge (92.9%) as per the statistical analysis plan.

**Table 9:** Adverse events following bronchial provocation and those not related to challenge in the safety population.

**System Organ Class**	**Preferred Term**	**1st Exc**^#^	**2nd Exc**	**Mann**^#^	**Meth**^#^	**NCR**^#^
n		435	429	419	420	436

**1.1 All**		25 (8%)	21 (7%)	130 (41%)	89 (28%)	49 (16%)

**Cardiac Disorders**	*Class Totals*	1 (5%)	0	6 (27%)	13 (59%)	2 (9%)
	
	Dizziness	1		5	13	0

**Gastrointestinal disorders**	*Class Totals*	1 (5%)	1 (5%)	14 (70%)	0	4 (20%)
	
	Nausea		1	5		1
	
	Retching	1		4		

**General disorders and administration site concerns**	*Class Totals*	1 (4%)	0	24 (86%)	3 (11%)	0
	
	Burning sensation mucosal			9		
	
	Feeling jittery			6	2	

**Nervous system disorders**	Class Totals	4 (20%)	2 (10%)	6 (30%)	4 (20%)	4 (20%)
	
	Headache	2	1	5	4	4

**Respiratory, thoracic and mediastinal disorders**	*Class Totals*	12 (6%)	11 (6%)	73 (39%)	66 (36%)	23 (12%)
	
	Chest discomfort	3	1	13	18	1
	
	Cough	2	1	9	8	4
	
	Dyspnoea	4	3	12	22	1
	
	Nasal congestion			1	4	3
	
	Pharyngolaryngeal pain	1		11	1	1
	
	Reversible airways obstruction		1	3	2	
	
	Rhinitis			3	1	1
	
	Rhinorrhea	2		8		1
	
	Wheezing		1	6	7	1

^#^Exc = Exercise; Mann = Mannitol; Meth = Methacholine; NCR = not challenge related

## Discussion

In these subjects with very mild symptoms suggestive of asthma and good lung function, sensitivity and specificity were equivalent for mannitol and methacholine to identify EIB and a clinical diagnosis of asthma. There were no serious adverse events associated with the tests and both were generally well tolerated.

Mannitol sensitivity to identify EIB was lower than that previously reported in subjects with a definite diagnosis of asthma [21,22]. The lower sensitivity of mannitol to identify EIB reported here is consistent with the mild EIB (50% of subjects had falls in FEV_1 _≤ 15%) documented in this group who did not have a definite diagnosis of asthma. The % fall in FEV_1 _in the asthmatics reported by Brannan [21] was 40 ± 19% (SD) and by Munoz [22] it was 37% ± 16% (SD). In the study of Holzer [23] a PD_15 _to mannitol had a sensitivity of 83% to identify EIB in a group of athletes with a 25.4% ± 15% (SD) fall in FEV_1 _after eucapnic voluntary hyperpnea, a surrogate challenge for EIB.

The sensitivity of mannitol to identify a Exc+ 20% fall in FEV_1 _was > 80% when those with a prolonged mannitol test time were excluded. Sensitivity of the mannitol to detect a Exc+10% fall was also > 80% in those with an asthma symptom severity score of 2 rather than 1, although the numbers with this score were smaller.

Sensitivity and specificity of methacholine and mannitol for a clinical diagnosis of asthma was equivalent in this study, but the values were lower than those reported in well-defined populations of asthmatic and healthy subjects [7] and similar to those reported for epidemiological studies [24-26]. In the children less than 18 yrs, however, mannitol had a 18.5% higher specificity (81.4% vs 62.9%) for identifying a clinical diagnosis of asthma and a 10% lower sensitivity (60.1% vs 70%) to identify EIB compared with methacholine.

This lower than expected sensitivity for mannitol and methacholine to identify EIB or a clinical diagnosis may be explained by the relatively unusual nature of this group of subjects who would not have qualified either for a clinical study on asthmatic subjects or a study on healthy subjects. The exclusion criteria required that the subjects not be symptomatic to the allergens to which they demonstrated atopy at the time of the study. This too is unusual and may affect mast cell number and IgE status. There was a lack of agreement between the clinical diagnosis and response to the challenge tests in 27% of the subjects; 15% of subjects given a diagnosis of asthma were negative to all three challenge tests and 12% with two or more positive challenge tests were deemed not to have an asthma diagnosis at Visit 5. The dose of mannitol (PD_15_) or concentration of methacholine (PC_20_) in those who were positive in this study was consistent with the values reported previously in clinically recognised asthmatics not taking inhaled corticosteroids [27,28].

The prevalence of BHR identified by the three tests differed by only 3.2% (from 41.6% to 44.8%). This suggests that the cut-off points used to define BHR were appropriate. Further, the range of severity of BHR was similar for all the tests with 50% being in the mild range for mannitol and exercise and 75% in the mild range for methacholine [2,29]. However it was not always the same subject responsive to all the BPTs and only 17.9% of subjects were positive to all three challenge tests. This probably reflects the natural variability of the test responses in people with mild BHR and intermittent symptoms.

Some long-held beliefs about methacholine were not upheld by the results of this study. Methacholine did not have a higher sensitivity than exercise and mannitol to identify BHR in this population, and the prevalence of BHR was similar for all tests. Methacholine did not have a high negative predictive value for a clinical diagnosis of asthma. There were 118 subjects negative to methacholine who were given a diagnosis of asthma at Visit 5. It was also not uncommon for methacholine to miss EIB, and 73 of the 163 subjects (45%) positive to exercise were negative to methacholine, confirming previous findings in young people with good lung function [30]. The sensitivity of a positive response to the first exercise test to predict a positive response to the second test (62%) was the same as the sensitivity of mannitol and methacholine to predict at least one positive test to exercise. This low sensitivity probably relates to the variability in the mild response to exercise, particularly in those without symptoms of allergy at the time of testing. By performing a second test, an extra 44 people were identified as having EIB. Care was taken in this study to minimise the potential for variability, and there was good overall agreement between the two exercise test results; however, this was primarily due to the number of negative exercise tests.

Only one exercise test and one time point of ≥ 10% was required to be the 'gold' standard used for BHR. This criterion may be interpreted by some investigators as not strict enough because of the variability of the response to exercise. However the value for sensitivity of mannitol to identify EIB was relatively unchanged (59.8% vs 58.3%) when only those subjects with two time points on one exercise challenge were ≥ 10%, or one time point was greater than ≥ 15%, were included in the analysis. The value for sensitivity for methacholine however rose from 55.3% to 63.1% applying the same criteria.

There were 7.5% of subjects with a positive response to bronchodilator at baseline who were not excluded at entry on the basis of having a > 95% chance of having asthma, presumably as the other evidence was not supportive. This group of 28 showed a higher value for sensitivity of mannitol (68.4% vs 59%) and methacholine (73.7% vs 56%) to identify EIB when compared with the entire population.

Cockcroft [31] has found that methacholine sensitivity to identify BHR of 4 mg/ml or more is lower using the dosimeter method compared with the tidal breathing method. The dosimeter method used here is one recommended in the American Thoracic Society guidelines that categorize BHR between 4–16 mg/ml as borderline [2], is in common use, and delivery of the aerosol by this method involves a similar inspired breathing pattern as the mannitol with which it was being compared. We challenged to 16 mg/ml and, on the basis of the Cockcroft observation, this may be equivalent to 8 mg/ml on the tidal breathing method. A PC_20 _of > 8 mg/ml is the value for normality illustrated in the 2007 GINA guidelines [32] and stated by the British Thoracic Society [33].

This study included steroid naïve subjects who were considered to have a greater than 5% and less than 95% probability of having asthma at entry based on spirometry, response to bronchodilator, symptoms, history and skin tests. The clinical value in comparing different diagnostic tools, such as the challenge tests used here, cannot really be judged because there is no gold standard test for the diagnosis of asthma. The strategy used here for a 'gold standard' for the diagnosis of asthma was to have a respiratory physician assign a diagnosis at Visit 5 on the basis of having all the information (including the exercise test results) except the results of the methacholine and mannitol tests. The information available was likely to be more than most clinicians would have available at assessment and thus as close to a clinical gold standard as one is likely to find. There was good agreement (71.6%) with the exercise test result and ClinDx5+ diagnosis of asthma with only 14 subjects positive to exercise being considered not to have asthma (ClinDx5-). This supports the fact that BHR to exercise, i.e. EIB, is accepted by clinicians as consistent with a diagnosis of asthma. The agreement between a ClinDx5 diagnosis and the methacholine result was 59.6% and mannitol was 61.8%.

In the Phase 3 study on mannitol, in subjects with known asthma and healthy subjects without asthma, the specificity and sensitivity of mannitol to identify a clinical diagnosis of asthma was 59.8% and 95.2% and it was 88.7% and 95% when those with a negative mannitol test being treated with inhaled corticosteroids were excluded [7]. In this study in subjects with symptoms but without a definite diagnosis of asthma at entry mannitol had a sensitivity and specificity of 56% and 73% and this was no different to methacholine 51% and 75%.

In conclusion, mannitol and methacholine provided therapeutic equivalence to identify BHR, EIB, and a clinical diagnosis of asthma in a group of subjects suspected of having asthma but without a clear diagnosis. While mannitol has potential to replace other challenges for BPT, any of these tests can provide specific information and insight regarding mechanism in a particular patient.

## Competing interests

SDA is the inventor of the mannitol test however the intellectual property is owned by her employer, the Sydney South West Area Health Service. SDA has not received any fees personally from Pharmaxis. SDA has undertaken research studies that were funded by Pharmaxis. She is a shareholder in Pharmaxis but holds no options. She is likely to benefit from royalties in the future.

BC is an employee and a shareholder of Pharmaxis Ltd.

JW is the President of, and is a shareholder in, CompleWare Corporation. CompleWare received a fee from Pharmaxis Ltd. for services in carrying out the clinical trial.

SN is the statistician employed by CompleWare and carried out the statistical analysis.

SS has received an honorarium from the sponsor of the trial (Pharmaxis Ltd) for chairing a pre-meeting seminar at the AAAAI conference and received a research grant for study participation from Pharmaxis.

DSP participated in the study through Colorado Allergy and Asthma Centers PC, which received a research grant for study participation from Pharmaxis Ltd.

There are no other competing interests or conflicts of interest.

## Authors' contributions

BC, JW, and SDA designed the study (after consultation with the FDA). CompleWare (which employs JW and SN) ran the clinical trial and the data acquisition, and wrote the report on which this paper is based. SN was the statistician for CompleWare. SS and DP were principal investigators who presented abstracts of the work at academic society meetings. All authors contributed suggestions and have approved the final version of the paper. SDA wrote the paper and prepared it for publication and made the decision to submit it to Respiratory Research.
